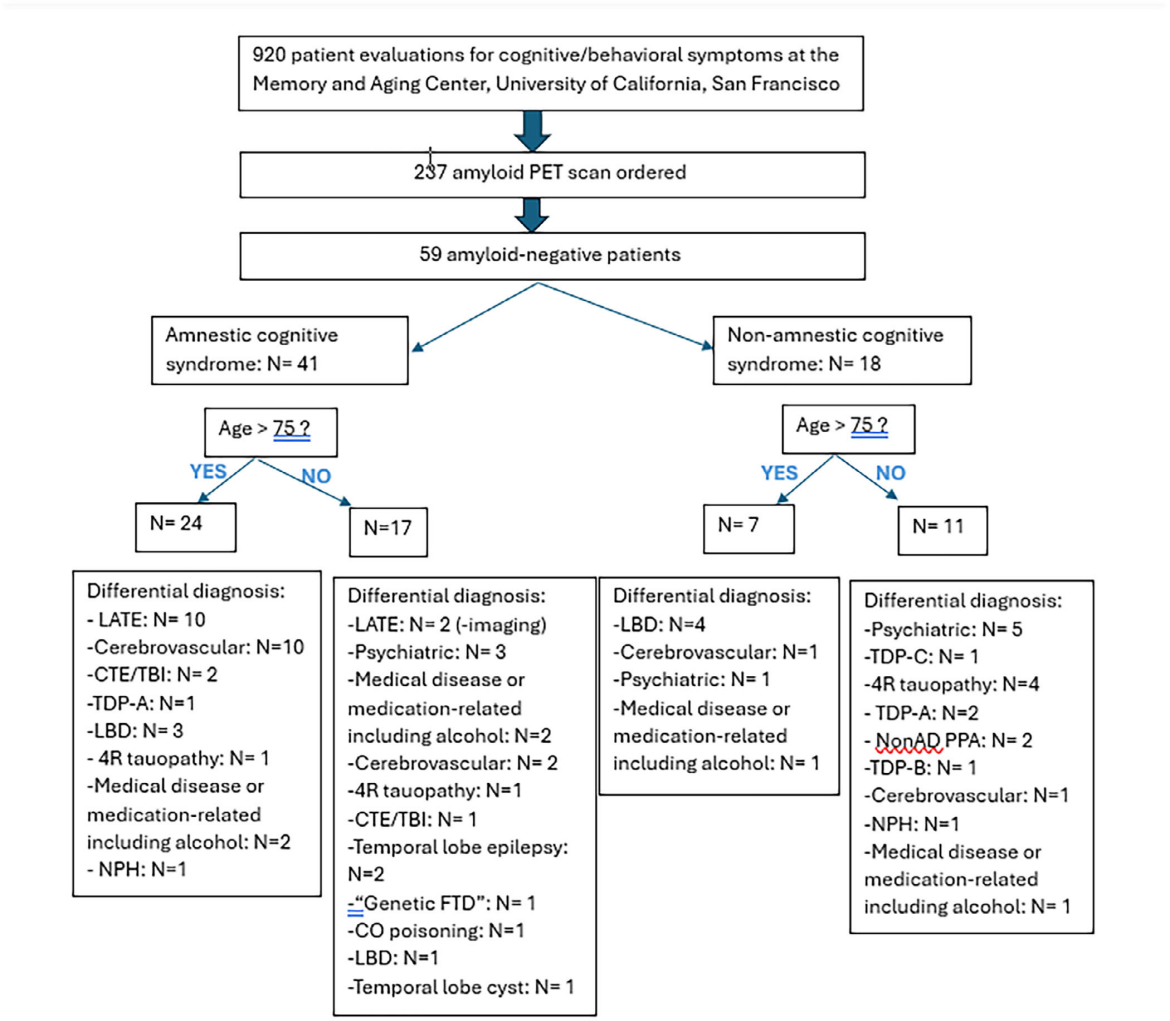# Case‐Based Review of Amyloid PET Negative Cases in a Large Memory Clinic: Considerations for Differential Diagnosis

**DOI:** 10.1002/alz70856_103882

**Published:** 2025-12-25

**Authors:** Manizhe Eslami‐Amirabadi, Tara N. Ellingson, Melanie Stephens, Bruce L. Miller, Gil D. Rabinovici, Lawren VandeVrede

**Affiliations:** ^1^ University of California, San Francisco, San Francisco, CA, USA; ^2^ Memory and Aging Center, San Francisco, CA, USA; ^3^ Weill Institute of Neuroscience, San Francisco, CA, USA; ^4^ Memory and Aging Center, Weill Institute for Neurosciences, University of California, San Francisco, San Francisco, CA, USA; ^5^ Global Brain Health Institute, University of California, San Francisco, San Francisco, CA, USA; ^6^ Memory and Aging Center, University of California San Francisco, San Francisco, CA, USA; ^7^ Department of Neurology, Memory and Aging Center, University of California San Francisco, San Francisco, CA, USA; ^8^ UCSF Alzheimer's Disease Research Center, San Francisco, CA, USA; ^9^ Memory and Aging Center, Weill Institute for Neurosciences, University of California, San Francisco (UCSF), San Francisco, CA, USA; ^10^ Memory and Aging Center, Weill Institute for Neurosciences, University of California San Francisco, San Francisco, CA, USA; ^11^ Molecular Biophysics and Integrated Bioimaging Division, Lawrence Berkeley National Laboratory, Berkeley, CA, USA; ^12^ University of California, Berkeley, Berkeley, CA, USA; ^13^ Department of Radiology and Biomedical Imaging, University of California San Francisco, San Francisco, CA, USA; ^14^ Lawrence Berkeley National Laboratory, Berkeley, CA, USA; ^15^ Weill Institute for Neurosciences, University of California, San Francisco, San Francisco, CA, USA; ^16^ Helen Wills Neuroscience Institute, University of California Berkeley, Berkeley, CA, USA; ^17^ Memory and Aging Center, UCSF Weill Institute for Neurosciences, University of California San Francisco, San Francisco, CA, USA; ^18^ University of California San Francisco, San Francisco, CA, USA

## Abstract

**Background:**

Diagnosis of Alzheimer's disease (AD) by the clinical criteria has limited diagnostic accuracy compared to pathology. Amyloid positron emission tomography (PET) imaging (noninvasively detects amyloid plaques, a core neuropathological feature of AD) had an important effect on diagnostic accuracy and clinical decision‐making for dementia patients. Medicare's decision to cover this test increased its utilization. Case‐based reviews of amyloid PET utilization and its effect on clinical care will inform our understanding of the real‐world differential diagnosis of dementia.

**Method:**

1708 patient encounters were performed at the Memory and Aging Center, University of California, San Francisco between July 2023 and July 2024 including 920 encounters for 547 unique patients with a dementia biomarker testing order (including PET scan and fluid biomarkers). For all patients evaluated by amyloid PET, charts were reviewed for differential diagnosis and imaging findings. We reviewed the diagnostic criteria for other dementia syndromes suggested by the provider to confirm its accuracy. Our protocol and results are summarized in Figure 1.

**Result:**

Amyloid PET was ordered for 237 (43%) but not performed in 59 (24%). 24% (57) of patients had a negative PET scan. Diagnoses for these patients included Limbic predominant age‐related TDP proteinopathy (LATE, *N* = 12), cerebrovascular disease (*N* = 14), Lewy body disease (*N* = 8), 4R tauopathies (*N* = 6), functional/psychiatric condition (*N* = 8), among others (*N* = 23). Many of the patients received more than one contributing diagnosis, especially the patients older than 75 years old.

**Conclusion:**

In the era of disease‐modifying treatments, amyloid PET will increasingly be used to identify patients appropriate for amyloid‐directed treatments, leading to continued diagnostic uncertainty for patients with a negative Alzheimer's disease biomarker, especially in non‐specialist settings. This has ethical implications for caring for these patients. Further research is needed to establish reliable biomarkers for other neuropathological diagnoses to reduce diagnostic uncertainty and improve the care for non‐Alzheimer's dementia.